# A New GPS SNR-based Combination Approach for Land Surface Snow Depth Monitoring

**DOI:** 10.1038/s41598-019-40456-2

**Published:** 2019-03-07

**Authors:** Wei Zhou, Lilong Liu, Liangke Huang, Yibin Yao, Jun Chen, Songqing Li

**Affiliations:** 1 College of Geomatic Engineering and Geoinformatics, Guilin University of Technology, Guilin, 541004 China; 2 Guangxi Key Laboratory of Spatial Information and Geomatics, Guilin, 541004 China; 3 GNSS Research Center, Wuhan University, Wuhan, 430079 China; 4 School of Geodesy and Geomatics, Wuhan University, Wuhan, 430079 China

## Abstract

Snow is not only a critical storage component in the hydrologic cycle but also an important data for climate research; however, snowfall observations are only sparsely available. Signal-to-noise ratio (SNR) has recently been applied for sensing snow depths. Most studies only consider either global positioning system (GPS) L1 or L2 SNR data. In the current study, a new snow depth estimation approach is proposed using multipath reflectometry and SNR combination of GPS triple frequency (i.e. L1, L2 and L5) signals. The SNR combination method describes the relationship between antenna height variation and spectral peak frequency. Snow depths are retrieved from the SNR combination data at YEL2 and KIRU sites and validated by comparing it with *in situ* observations. The elevation angle ranges from 5° to 25°. The correlations for the two sites are 0.99 and 0.97. The performance of the new approach is assessed by comparing it with existing models. The proposed approach presents a high correlation of 0.95 and an accuracy (in terms of Root Mean Square Error) improvement of over 30%. Findings indicate that the new approach could potentially be applied to monitor snow depths and may serve as a reference for building multi-system and multi-frequency global navigation satellite system reflectometry models.

## Introduction

Snow is an essential element that affects the global climate in the cryosphere, which plays a crucial role in surface energy balance and global climate change feedback^1^. Water runoff from snowmelt is an important freshwater resource that can identify possible variation trends and predict the effects of snow situations on local freshwater availability and flood through the measurement of snow depths^2^. However, existing automated techniques, such as sonic depth and infrared optical measurements, present high temporal resolution, and information on *in situ* spatial height variations is lacking^3,4^. Thus, these methods cannot easily meet the demands of monitoring snow depth variations primarily due to the high temporal–spatial variability.

The global positioning system (GPS) is widely applied in navigation, timing and positioning and has been fully operational since 1994^5^. In addition to the fundamental applications, GPS can be used to retrieve surface characteristic parameters on a global scale with minimal cost. The physical reflectivity and polarisation characteristics of GPS-reflected signals on surfaces are used to infer physical properties^6,7^. This measurement concept is known as GPS reflectometry (GPS-R), which has been used in sea surface roughness and altimetry, sea wind, sea ice, soil moisture and vegetation biomass^8–17^. The GPS-R technology offers numerous advantages, such as high spatiotemporal resolution, high precision and adaptability to all weather conditions. Moreover, GPS data are continuous and can be freely obtained from the International GNSS Service (IGS) network. Therefore, GPS-R is an effective method for estimating long-term land surface parameters. Bondi *et al*.^18^ first proposed the concept of GPS-R, that is, the static GPS-based scatterometry technique for remote sensing. The development of GPS-R is aimed at taking sea surface altimetry measurements using GPS-reflected signals^19^. Altimetry information is successfully obtained and shows the coherent effects between direct and reflected signals. Comp *et al*.^20^ and Bilich *et al*.^21^ determined the mapping relationship between the signal-to-noise ratio (SNR) data of GPS-reflected signals with different frequencies and the multipath effects and then established an altimetry model. The GPS phase and SNR models are mainly used to detect sea surface parameters. GPS-R has been applied to sensing of land surface characteristic parameters, including snow depth retrieval^22,23^, with improvements in GPS-R theory. Larson *et al*.^24–26^ first measured soil moisture using GPS multipath signals from the Continuously Operating Reference Station network and demonstrated that the amplitude and phase of GPS-reflected signals change with soil moisture variations around the GPS antenna. Surface snow depths are successfully estimated using the same SNR detection method, which consists of direct and reflected signal components^27,28^. The situ measurements and the retrieved snow depths and soil moisture show a good agreement. Considerable efforts have been exerted to sense snow depth parameters using GPS/GNSS measurements. A physical-based multipath forward model was established^26^ to investigate the effects of varying coherence on SNR observations. This model considers the polarisation of the received GPS signals and response characteristics between the GPS antenna and the ground-based GPS-R techniques. As previously mentioned in the forward model, the SNR observations of GPS L1-, L2C-, L2P- and L5-reflected signals have been developed and used to estimate snowstorm depths^4,29–31^. These methods use the multipath SNR data caused by the interference between direct and reflected signals. The results show weak deviations and high correlation relative to the *in situ* observations.

Numerous studies have paid increasing attention to the combination of the phase and SNR measurements of GNSS signals to improve the performance of existing GNSS-R models. Ozeki *et al*.^32^ proposed a dual-frequency phase combination (termed L4 phase) from GPS L1 and L2 signals which are normally used to limit geometric parameters, eliminate ionospheric delay errors and implement forward scattering. This L4 method offsets the flaws of conventional altimetry detection models and obtains similar results to GPS SNR data. In addition to the study on the GPS L4 model, the studies on the SNR and L4 observations of GLONASS showed no notable differences^33^. Considering the excellent performance of GPS L5 in retrieving snow depth, Yu *et al*.^34^ first demonstrated that the snow depth retrieved from a linear combination of phase observations of GPS triple-frequency (i.e. L1, L2 and L5) signals showed an improvement relative to existing methods. Therefore, several studies have preliminarily indicated that the accuracy of detecting snow depth can be improved using a combination of GNSS signals.

An improvement of snow depth with 10%–20% uncertainties will greatly contribute to hydrological and climatological studies considering the problems of conventional snow depth^27^. Existing SNR detection methods can be independently applied for each carrier, but their performance largely depends on the quality and quantity of SNR observations. Thus, the current work considers GPS triple-frequency reflected signals in modelling to increase the valid SNR observations and decrease the random errors. A new snow depth estimation approach is then proposed using the SNR combination of GPS triple-frequency (i.e. L1, L2 and L5) signals in multipath effects. Finally, the performance of the new approach is tested using *in situ* snow depth observations at YEL2 and KIRU sites, and the conclusion is drawn.

## Theory and Methods

### Snow Depth Retrieval

**Figure 1:**
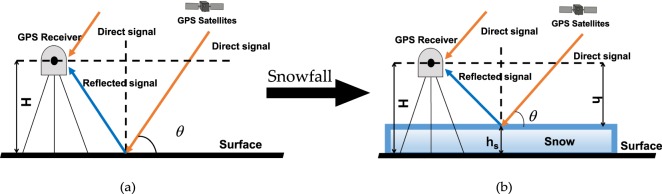
Geometry of GPS-R technique. H denotes the antenna height above the ground without snow coverage, h denotes the antenna height from the reflection surface, and ${h}_{s}=H-h$ can obtain the snow depth. (**a**) Snow-free; (**b**) covered with snow.

Figure 1 illustrates two cases in which direct and reflected signals arrive at the zenith-looking GPS antenna when the surface is snow-free and covered with snow of a certain depth, respectively. The surface roughness increases and causes diffused reflection when the land surface is covered with snow. Meanwhile, strong specular reflection occurs. Most scattering processes only occur on the snow surface when direct signals reach the snow surface. For snow around the antenna, the antenna height above the surface is shortened by a length equal to the snow depth. If the initial GPS antenna height is known in advance and the antenna height above the snow surface can be estimated, then snow depth can be obtained. This basic idea is used in GPS-R-based snow depth estimation methods^34^. Reflected signals have one excess time delay compared with direct signals and are also called GPS multipath. These signals are also related to antenna height. The amplitude characteristics of reflected signals produce changes due to the effect of snow surface media. The information of surface snow depth is obtained by measuring the characteristics.

**Figure 2:**
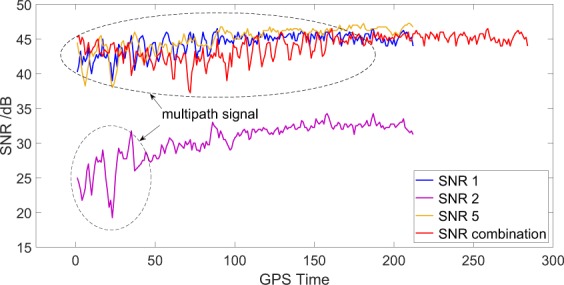
Observed series of GPS L1, L2, L5 and the SNR combination of GPS triple-frequency signals (transformed to linear scale) for ascending satellite PRN03 (low satellite elevation angles) for one successive day at YEL2. The signal oscillations are affected by multipath effects.

Generally, surface characteristic parameters can be estimated using particular GPS receivers with two antennas, namely, left-hand circular polarised (LHCP) and right-hand circular polarised (RHCP) antennas, which receive direct and reflected signals, respectively^35,36^. These antennas use GPS-reflected signals for remote sensing on the basis of the waveform correlation. However, the high cost of dual-antenna-mode GPS receivers limits the expansion of GPS-R. Therefore, a single-antenna receiver has been developed for observation and experiment. The multipath effects of a receiver can be diminished by choke coil and demagnetisation plate but cannot be completely eliminated. The polarisation of GPS-reflected signals at low elevation angles easily interferes with direct signals after entering the receiver, thereby causing signal oscillation (Fig. 2). Snow depth estimation with a geodetic receiver mainly uses the signal oscillation resulting from the multipath effect. As shown in Fig. 2, a high similar tendency exists for GPS L1, L2, L5 and the SNR combination from the GPS. However, the signal oscillations of the SNR combination are more continuous than those of the remaining SNR observations.

SNR is one of the main GPS observations for assessing the signal quality and noise characteristics of typical GPS measurements^12,37^. The composite signals received by a receiver include direct and reflected signals through a surface medium (Fig. 2). Without multipath effects, SNR observations smoothly rise with the increase in elevation angles. The direct component shows a long-term and stable trend, and the reflected one demonstrates partial oscillation (within the dotted box of Fig. 2). The effect of the reflected signal considerably exceeds that of the direct one. Consequently, the direct signals for a long time series can be fitted by the low-order polynomial method, and the detrended SNR (dSNR) can be obtained by making a difference between the composite SNR signals and the fitted values.

### GPS data issues

The scattered GPS signals from the land surface around the receiver antenna interfere with the direct signals, thus contributing to the measured phase, power and amplitude^38^. Snow depth sensing utilises SNR observations. Multipath effects can more conveniently be extracted from GPS SNR observations than from carrier-phase and code observations. GPS SNR data from geodetic networks are found in Receiver Independent Exchange Format (RINEX) observation files. Many network operators also archive the so-called S1 and S2 observables, referred to as signal strength in RINEX specifications. S1/S2 in Standardised GPS RINEX corresponds to the quantity called carrier-to-noise density ratio (C/N0), that is, the ratio of signal power to the noise power spectral density^39^. For simplicity, S1/S2 observations are reported in the current work as SNR. Instantaneous SNR is described on the basis of Nievinski and Larson^40,41^ as follows:

1 $${\rm{SNR}}=({S}_{d}+{S}_{r}+{S}^{I}){S}_{n}^{-1}+2{S}_{n}^{-1}\sqrt{{S}_{d}{S}_{r}}\,\cos \,\phi ,$$

where ${S}_{d}$ and ${S}_{r}$ are the powers of the direct and reflected signals, respectively; ${S}_{n}$ is the noise power; and ${S}^{I}\,\,$is the incoherent power. The incoherent component is the sum of the direct and reflected powers (although direct incoherent power is negligible); it is denoted as ${S}^{I}={S}_{d}^{I}+{S}_{r}^{I}\approx {S}_{r}^{I}$. Assuming a horizontal reflecting surface, the interferometric phase $\phi$ is written as follows:

2 $$\phi =\frac{4\pi h}{\lambda }\,\sin \,\theta +\varphi $$

where $h$ is the reflector height (i.e. the vertical distance from the phase centre of the GNSS antenna to the reflecting surface), $\theta$ is the elevation angle of the satellite, $\lambda$ is the signal wavelength and $\varphi$ denotes the phase contribution of the antenna pattern and electromagnetic properties of the reflecting surface.

**Figure 3:**
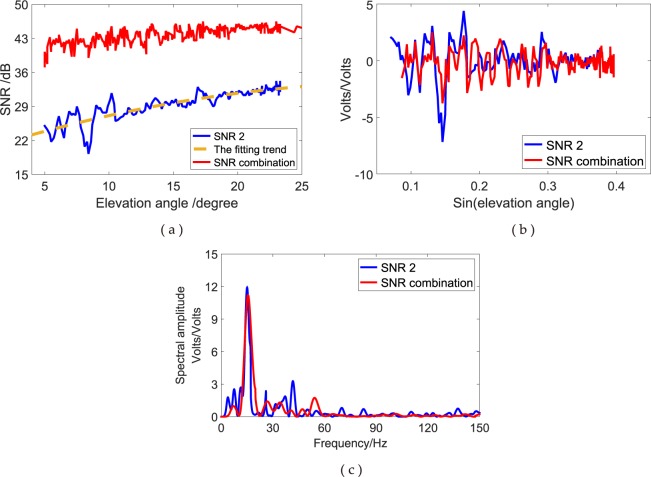
L2 SNR observations and SNR combination of GPS triple-frequency signals, multipath pattern and Lomb–Scargle (L–S) periodogram for PRN 03 satellite at YEL2 site. (**a**) Triple-frequency SNR combination and L2 SNR observations; (**b**) multipath pattern after detrending; (**c**) L–S periodogram of the multipath pattern.

The SNR magnitude in low elevation angles increases or decreases with the changes in the amplitudes or the interferometric phase (Fig. 3a). The forward model describes the function of the observed SNR, modelled as the sum of a trend SNR (tSNR) and detrended interference fringes (dSNR), to improve the development of near-surface reflectometry. In Equation (1), $({S}_{d}+{S}_{r}+{{\rm{S}}}^{I}){S}_{n}^{-1}$ is the direct trend and should be removed, and $2{S}_{n}^{-1}\sqrt{{S}_{d}{S}_{r}}cos\phi$ denotes the interference fringes that cause the signal oscillations for the SNR observations. The oscillation, also called multipath effect, is the interference between the direct and reflected signals simultaneously received by GPS receivers. For GPS-R, the incoherent ${S}^{I}$ and coherent ${S}_{d}+{S}_{r}$ powers without the interference are useless.

The SNR observations from each GPS carrier are independently calculated with each track separated into ascending and descending paths. According to Equation (1), the SNR observations of GPS triple-frequency signals can be obtained and are usually denoted as ${{\rm{SNR}}}_{1}$, ${{\rm{SNR}}}_{2}$ and ${{\rm{SNR}}}_{5}$. The proposed approach utilises the SNR combination of GPS triple-frequency (i.e. L1, L2 and L5) signals. The obtained SNR observations in the same form are merged into a large aggregate called ${{\rm{SNR}}}_{com}$ observation. Then, the modelling of the relationship amongst ${{\rm{SNR}}}_{1}$, ${{\rm{SNR}}}_{2}$, ${{\rm{SNR}}}_{5}$ and triple-frequency SNR combination is described as follows:

3 $$SN{R}_{com,i}=[SN{R}_{1,i}\,SN{R}_{2,i}\,SN{R}_{5,i}],$$

where ${{\rm{SNR}}}_{com}$ denotes the observed SNR combination of GPS triple-frequency (i.e. L1, L2 and L5) signals; *i* = 1, 2, 3, …, 364, 365; and ${{\rm{SNR}}}_{1}$, ${{\rm{SNR}}}_{2}$ and ${{\rm{SNR}}}_{5}$ are the SNR observations of GPS L1, L2 and L5 signals, respectively. As previously noted in Equation (3), ${{\rm{SNR}}}_{com}$, a new form of the observed SNR, is expressed in the form of the SNR combination of the received GPS triple-frequency signals.

Isolating the last item from Equation (3) by removing the direct trend using a low-order polynomial detrending is necessary to solve for reflector height (${h}_{s}$). The dB scale is converted into a linear one, such as volts for linearity, using the equation ${\rm{SNR}}(\frac{Volts}{Volts})={10}^{\frac{SNR(dBHz)}{20}}$^4^. After removing the direct trend, the multipath patterns of GPS signals (termed dSNR) are obtained (Fig. 3b). dSNR represents the effects of the reflected signals on the observed SNR signals; it can be represented by a quasi-cosine function, as shown as follows^42^:

4 $${{\rm{dSNR}}}_{com}={{\rm{SNR}}}_{com}-({S}_{d}+{S}_{r}+{S}_{r}^{I}){S}_{n}^{-1}=A\,\cos (\frac{4{\rm{\pi }}h\,\sin \,\theta }{\lambda }+\varphi ),$$

where $A$ is the amplitude and $\varphi$ is the phase. Suppose that the antenna height (*h*) is consistent during the period of GPS satellites, the reflected surface is horizontal and the frequency domain of the multipath pattern is a constant with respect to the sines of the satellite elevation angle. Equation (4) can be simplified into a standard cosine function by assuming that $t=\,\sin \,\theta$ and $f=2h/\lambda$, as expressed as follows:

5 $${{\rm{dSNR}}}_{com}=A\,\cos (2{\rm{\pi }}f{\rm{t}}+\varphi ),$$

where frequency domain (*f*) contains the antenna height from the reflection surface (*h*).

The spectrum analysis of Equation (5) can yield the frequency domain (*f*). Generally, the fast Fourier transform (FFT) algorithm can be used to solve the uniform sampling problems in the frequency domain. However, using the FFT algorithm to sample the SNR signals in the integral period is difficult, and a non-interval method is essential for resampling dSNR data. Therefore, the L–S Fourier transform method^11^ can be used to sample ${{\rm{dSNR}}}_{com}$; this method not only effectively extracts weak periodic signals from the time domain sequence but also substantially weakens the false signals generated by the inhomogeneity of the time domain sequence to a certain extent. With these advantages, the frequency domain (*f*) of the reflected signals from the GPS multipath pattern can be obtained by performing the L–S spectrum analysis on the ${{\rm{dSNR}}}_{com}$. The results show no evident differences in the amplitudes of L2 SNR and triple-frequency SNR combination observations (Fig. 3c). The frequency domain of the multipath pattern is related to the antenna height (*h*); thus, the relationship can be written as follows:

6 $$h=\frac{\lambda f}{2}.$$

The antenna height (*h*) changes with the height variations of the land surface covered by thick snow, and the frequency domain of the multipath pattern is affected by snow depth variations. In addition, the antenna height (*H*) under snow-free condition is known in advance; thus, the snow depth estimations (${h}_{s}$) can be obtained by ${h}_{s}=H-h$.

The snow depth estimation method using SNR data relies on a statistical analysis model. The systematic and random errors and differences in the physical properties of snow at different times, elevation angles and reflection points are seldom considered. Therefore, the estimation accuracy of snow depth is improved in this study by mainly increasing SNR observations, controlling observation quality and analysing estimation errors. The main procedures of estimating snow depth from GPS triple-frequency SNR data can be summarised as follows:

1. The SNR data of GPS L1, L2 and L5 carriers are calculated, and the SNR observations of GPS triple-frequency signals are combined to obtain SNR_*com*_ observations.
2. A low-order polynomial function is used to separate the composite SNR_*com*_ data, and the tSNR_*com*_ and dSNR_*com*_ components are obtained.
3. The dSNR_*com*_ component, which is analysed by using the L–S periodogram, is resampled and obtained.
4. The frequency *f* is converted to the reflected height *h*_*s*_.

## Data Introduction

### GPS observations

**Figure 4:**
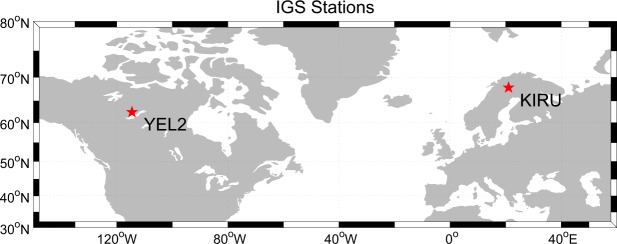
Location of GPS observatory stations. YEL2 and KIRU observatory stations are located in Yellowknife, Canada; and Kiruna, Sweden, respectively. This figure is drawn using MATLAB software.

Formed in 1994, the IGS aims to openly access and provide high-quality GNSS data products from several permanent GPS stations since 1994. The GPS, which was developed in the early 1960s, provides global and regional positioning, navigation and timing. The observations of GNSS observatory stations provided by the multi-GNSS experiments (MGEX), which can receive GPS triple-frequency observations, are few; thus, we select only two GNSS observatory stations provided by MGEX in Canada and Sweden with available co-located *in situ* snow depth measurements (Fig. 4): KIRU and YEL2 sites (http://www.igs.org/igsnetwork/). The receivers at the YEL2 and KIRU sites overlook the horizontal pristine snow area; their antenna heights are 1.0 and 1.8 m, respectively. The GNSS observatory stations are flat and covered with thick snow in the winter, and the reflector surface can be regarded as a horizontal plane. The minimum satellite elevation angle of the selected reflection signal is 5° when the local surface is flat according to the position relationship between the satellite/ground and the ground gradient. The sampling rate of the GPS triple-frequency (i.e. L1, L2 and L5) SNR data is 15 s. The GPS observations at the KIRU and YEL2 stations cover July 2015 to May 2016 and August 2015 to June 2016, respectively.

### Climate data

The *in situ* snow depth data are obtained from the National Climatic Data Center (https://www.ncdc.noaa.gov/); such data can provide the average snow depth measurements with a time resolution of one day. The distance between the GPS observatory stations and the *in situ* observations is close, and the *in situ* snow depths are treated as a reference. The co-located climate stations of the KIRU and YEL2 sites are Esrange and Yellowknife Henderson, respectively. Table 1 shows the basic parameters of the GPS observatory and climate stations, which ensure that the GPS observations and *in situ* snow depths are simultaneously monitored at a nearby place.

**Table 1:** Basic parameters of GPS observatory and climate stations.

Station name	Longitude/°	Latitude/°	Elevation/m	Antenna	Distance /km
KIRU	20.97	67.86	391.1	SEPCHOKE_MC	5.0
ESRANGE	21.07	67.89	335.0	—	
YEL2	−114.38	62.45	181.0	LEIAR25.R4	6.0
Y-H	−114.48	62.48	200.0	—	

### Results and analysis

Only two data sets of observations from the GPS satellite are used to test the proposed snow depth estimation approach because of few GPS stations tracking the L5 signal. The data sets are first briefly described, and their performances are then evaluated and compared. The GPS SNR observations (i.e. L1, L2 or L5) and SNR combination of GPS triple-frequency signals are used to estimate the snow depths and are compared with *in situ* snow depth measurements for one year from the two GPS sites.

### Characteristic Analysis

The modelled L1, L2 and L5 SNR observations are evaluated according to the multipath simulator presented by Nievinski and Larson^43^. The multipath simulator considers the coherence between direct and reflected signals and combinations of antenna and surface responses. Table 2 summarises all properties of the legacy to determine the differences amongst L1, L2 and L5 signals and modernised GPS signals that are relevant for the analysis.

**Table 2:** Comparison of GPS L1, L2 and L5 signals.

GPS signal	Observation	Wavelength (cm)	Frequency (MHz)	Chipping rate (Mchip/s)	Code length (chip)	Min received power (dBW)
L1 C/A	SNR1	19.0	154 × 10.23	1.023	1023	−158.5
L2P	SNR2	24.4	120 × 10.23	10.23	6.187 × 10^12^	−164.5
L5	SNR5	25.5	115 × 10.23	10.23	10230	−154.9

GPS satellites have been transmitting civilian access (C/A) and encrypted codes on L1 and L2 frequencies, respectively, since 1978. These codes can be obtained in all GPS sites, and they are called L1C/A and L2P. The new GPS L5 signals, which consist of two bi-phase shift key components in phase quadrature, can improve tracking sensitivity and measurement accuracy. This improvement can help us overcome the inherent weaknesses of the C/A signal and (semi-) codeless tracking of the encrypted P(Y) code. The new L5 has two codes (i.e. I5 and Q5) that include a data-free channel (Q5). No military signal is present for L5, and the separation of the above-mentioned two codes is achieved through phase quadrature. The dataless signals (L5–Q5) can improve the signal acquisition and tracking performance of receivers. Such an enhancement is particularly helpful in operations under low-SNR environments.

Chipping rate is an important parameter of code modulation to be considered in a multipath investigation^31^. This interplay between code chipping rate and interferometric delay affects the amount of interferometric power resulting from the receiver correlation of a clean replica against the recorded multipath (direct and reflected) signals. Furthermore, code length affects SNR through the amount of cross-channel self-interference. As the length of the C/A code is the shortest amongst the GPS L1C/A, L2P and L5 signals, the latter two are the less susceptible to this problem.

**Figure 5:**
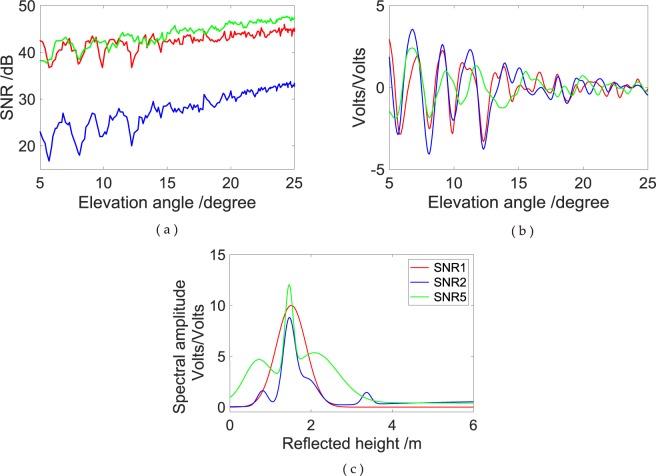
Comparison of simulated SNR1, SNR2 and SNR5 SNR observations at YEL2. (**a**) Simulated SNR of GPS triple-frequency signals from satellite PRN 06; (**b**) multipath modulations; (**c**) L–S periodogram of multipath pattern.

**Figure 6:**
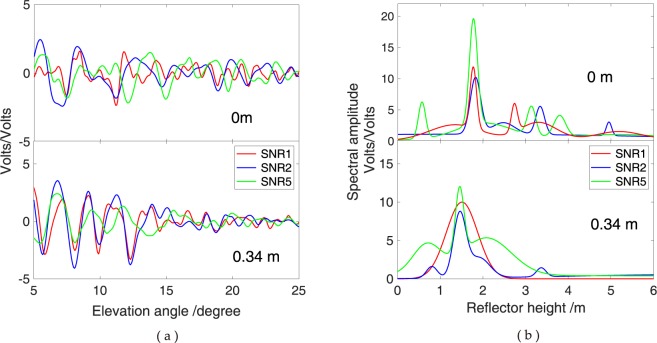
Effects of snow depth height on oscillation fringe visibility and amplitude power at GPS L1, L2 and L5 carriers for a geodetic antenna at the YEL2 site; labels refer to 0 and 0.340 m snow height values. (**a**) Detrended SNR observations; (**b**) L–S periodogram of dSNR observations.

SNR is the ratio of the signal to the noise power; thus, the minimum power is also one of the important differences. The received low minimum power results in the poor strength of L2P SNR measurements. The L2P SNR is approximately 15 dB, which is weaker than that of the L1C/A and L5 signals. Oscillations still exist in GPS SNR measurements despite the weak strength. These oscillations are shown in Fig. 5a. After removing the direct trend, low amplitude modulations can still be clearly seen in Fig. 5b, in which the elevation angle axis is linear with the interferometric delay. The L5 exhibits a fast chipping rate, thereby making the main peak in the auto-correlation function sharp. Furthermore, L5 has a minimum specified received power greater than that of L1 C/A and L2P; therefore, it would be expected to offer better tracking noise performance. As shown in Fig. 5c, an evident difference exists in the amplitude peaks from the frequency of modulations due to different signal strengths. Figure 6 shows the comparison of L1C/A, L2P and L5 SNR measurements from satellite PRN 06 at the YEL2 site.

In addition, the effects of changes in antenna height on the interferometric delay and power are considered. Figure 6 provides oscillation fringe visibility and amplitude power for surface snow depth measured upright at heights of 0 and 0.340 m. The reflections are assumed to occur on a horizontal surface with negligible roughness. In this setup, an increasing number of oscillation fringe cycles are observed as the satellite rises at the horizon mask to zenith with increasing snow depth when the antenna height is constant (Fig. 6a). Meanwhile, signal power changes are also evident. For the ordinary antenna, L5 is the strongest whilst L1 and L2 exhibit similar power. In the frequency domain, the peak amplitude of the SNR modulations evidently decreases with the increase of snow height (Fig. 6b). This phenomenon shows that a strong mirror reflection weakens because the diffuse scatter plays a major role when snow height increases.

### Single-frequency SNR Retrievals

**Figure 7:**
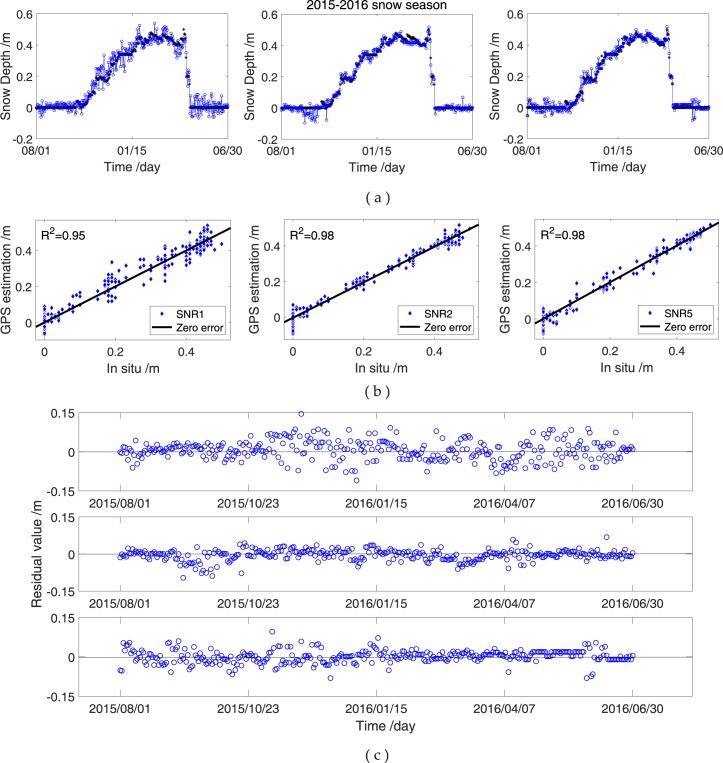
Comparison of SNR data of GPS L1, L2 and L5 signals and *in situ* snow depths at YEL2. (**a**) Snow depths estimated using SNR observations of GPS L1, L2 and L5 signals; (**b**) correlations between GPS L1, L2 and L5 observations and *in situ* measurements; (**c**) residuals between the estimated (from L1, L2 and L5 observations) and *in situ* snow depths.

**Figure 8:**
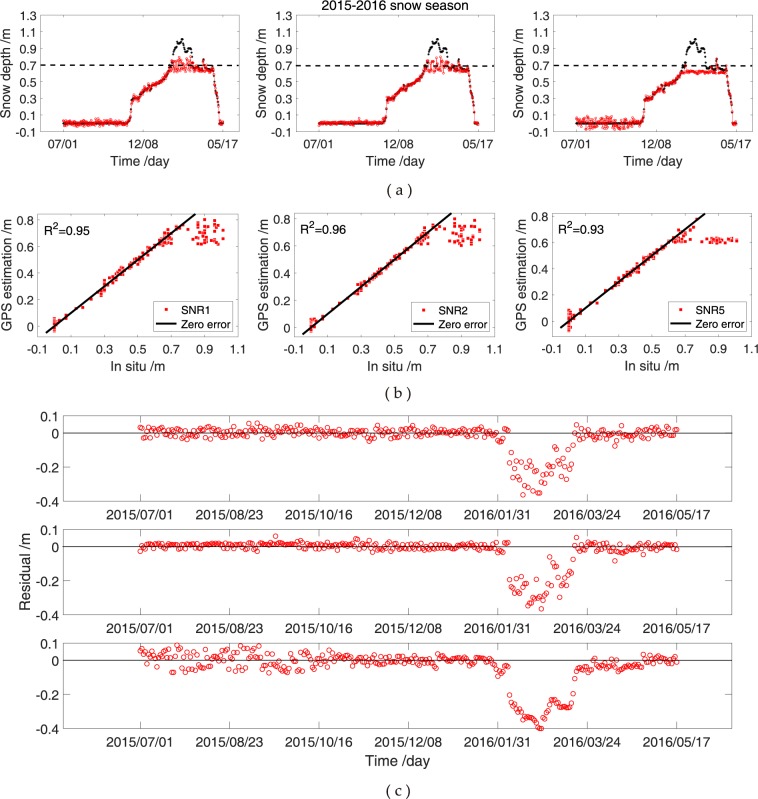
Comparison of SNR data of GPS L1, L2 and L5 signals and *in situ* snow depths at KIRU. (**a**) Snow depths estimated using SNR observations of GPS L1, L2 and L5 signals; (**b**) correlations between GPS L1, L2 and L5 observations and *in situ* measurements; (**c**) residuals between the estimated (from GPS L1, L2 and L5 observations) and *in situ* snow depths.

The GPS SNR observations are processed in accordance with the procedure presented in Equations (1–6). Not all SNR observations are useful, and only the peak amplitudes with several times of the background noise can be retained. Therefore, a new approach is proposed by combining the SNR data of GPS triple-frequency signals to increase the effective SNR observations and further improve the accuracy of snow depth estimation. Each effective SNR observation is computed using the L–S periodogram method to obtain GPS antenna height and snow depths. The reflected heights (*h*) amongst winter and summer days are used to estimate the snow-covered and snow-free ground height. The daily snow depth is the average value of all depths from each valid GPS SNR signal. Figures 7 and 8 show the comparisons of the GPS-estimated snow depths from SNR1, SNR2 or SNR5 data and *in situ* measurements at YEL2 and KIRU, respectively. Although the experiments using any of the GPS L1, L2 and L5 signals to estimate snow depths have been effectively conducted, completing and improving them remains essential because of the differences in GPS observatory stations.

At YEL2, snow depths estimated from the GPS L1, L2 or L5 signals alone are slightly different from the *in situ* snow depths in the winter of 2015, but the variations and trends are the same (Fig. 7a). The estimations of the aforementioned three SNR methods and *in situ* measurements demonstrate correlation coefficients of above 0.95. Thus, the estimation results show an excellent agreement with the *in situ* measurements (Fig. 7b). Meanwhile, the changes in snow depths are similar in the SNR combination observations and *in situ* measurements, and the estimations from the GPS L1, L2 or L5 SNR signals in snow-free condition illustrate a more evident deviation than those under thick snow conditions in 2015–2016 (Fig. 7c). This result could be due to the penetration of the L-band signal through the media. Figure 8 shows the snow depth estimations from the GPS L1, L2 or L5 SNR at the KIRU site. The estimated and *in situ* snow depths present a peak height in the spring of 2016, after which the snow gradually melts (Fig. 8a). Here, the correlation coefficients at KIRU are slightly lower than those at YEL2, and an evident range of the large residuals is observed at KIRU in the spring of 2016 (Fig. 8b,c). This occurrence is due to the largest depth of snow coverage at KIRU, which reached 1.1 m. Obtaining an accurate snow depth estimation with the aforementioned height is difficult because of the 1 m GPS antenna (Fig. 8a). Therefore, snow depth estimations are compared with *in situ* snow depths in the spring of 2016. Results show wide differences, and the SNR data are found to be unsuitable for estimating snow depths at the moment. The snow depth estimation at the KIRU site from 38 DOY to 73 DOY in 2016 is removed to reduce the influence on the results. At the YEL2 and KIRU sites, the estimation from the GPS L1, L2 or L5 signals exhibits the same trend, and the correlation coefficients are over 0.90 with the *in situ* measurements. Moreover, the L2 SNR for snow depth estimation is superior to the other two SNR methods, with L2-RMSE values of 2.6 and 2.2 cm at YEL2 and KIRU, respectively (Table 3). The differences in snow depth estimation may also be affected by other factors, such as wind and temperature, which cause variations. Although the correlation coefficient and RMSE are an imperfect coherence for YEL2 and KIRU, GPS SNR and *in situ* measurements almost show a good agreement.

**Table 3:** Correlation and RMSE between the snow depth estimations of the SNR data of GPS L1-, L2- or L5-reflected signals and *in situ* observations.

Snow season	GNSS station	Observations	ME/cm	RMSE/cm	*R* ^2^
2015–2016	YEL2	SNR1	4.3	4.0	0.95
		SNR2	2.9	2.6	0.98
		SNR5	3.0	2.5	0.98
	KIRU	SNR1	3.0	2.9	0.95
		SNR2	2.6	2.2	0.96
		SNR5	3.0	3.8	0.93

### Triple-frequency SNR Combination Retrievals

**Figure 9:**
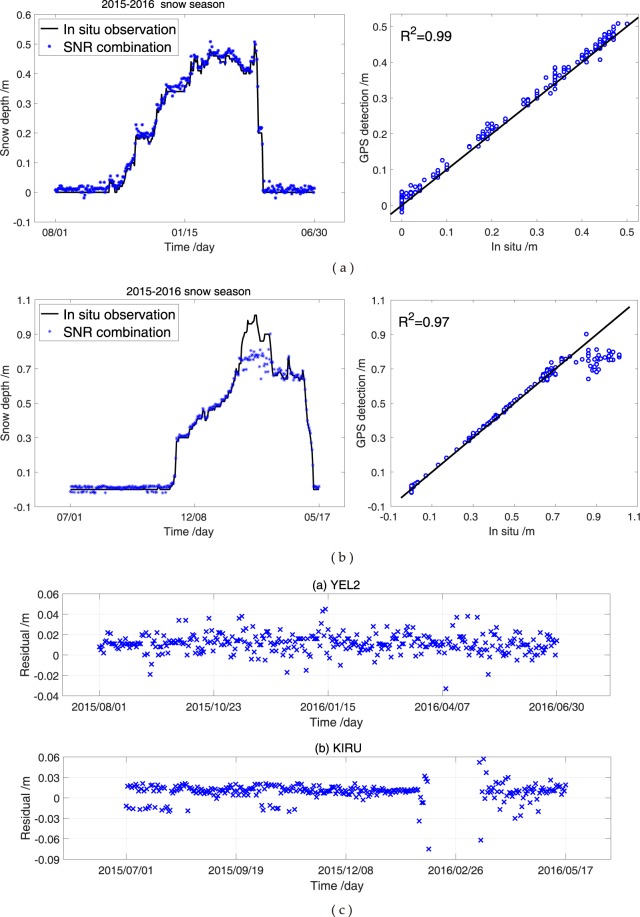
Comparison of SNR combination of GPS triple-frequency signals and *in situ* snow depths at YEL2 and KIRU. (**a**) YEL2; (**b**) KIRU; (**c**) residuals at YEL2 and KIRU.

Approximately 10 visible GPS satellites exist every epoch at YEL2 and KIRU. If the GPS triple-frequency (i.e. L1, L2 and L5) observations are included, considerable multipath effects exist. Moreover, numerous reflection points are observed on the ground and thus indicate that many sensing areas around the antenna are covered. Figure 9 shows the comparison of snow depth estimation from the combination of GPS triple-frequency signals and *in situ* measurements in the 2015–2016 snow season at YEL2 and KIRU. The results of both GPS stations show an excellent agreement with the *in situ* measurements (Fig. 9a,b). The snow depth estimation of the combination of GPS triple-frequency signals demonstrates a peak height in February 2016, similar to the *in situ* measurements. During the snow season, the estimations from GPS L1, L2 and L5 SNR illustrate an excellent agreement in comparison with the estimations under snow-free conditions. This phenomenon is also noted in the results of the SNR combination of GPS triple-frequency signals. Furthermore, the differences between snow depths are estimated using the combination of GPS triple-frequency signals; the *in situ* measurements decreased in the above-mentioned two cases. Thus, the SNR combination method is highly effective in snow depth estimation as the number of SNR observations increases. The existing deviations are due to the significant differences in the *in situ* and GPS observations, which are only considered as references. Moreover, the period used to estimate snow-free reflected heights is from beginning to end of the snow season. The assumed snow-free reflected heights are also inaccurate and affect the results due to the incompletely flat surface. For the SNR combination of GPS triple-frequency signals, the correlation coefficients for YEL2 and KIRU are 0.99 and 0.97, and the RMSEs are 1.5 and l.6 cm, respectively (Table 4). The residuals between the estimations from the SNR combination of GPS triple-frequency signals and *in situ* measurements in 2015–2016 snow season are analysed, and the results show that most of the residuals are between −4 and 4 cm (Fig. 9c). Some days with residuals of above 0.1 m are normal.

**Table 4:** Correlation and RMSE between the estimations of the SNR combination of GPS triple-frequency signals and *in situ* observations.

Snow season	GNSS station	ME/cm	RMSE/cm	*R* ^2^
2015–2016	YEL2	1.4	1.5	0.99
	KIRU	1.4	1.6	0.97

**Figure 10:**
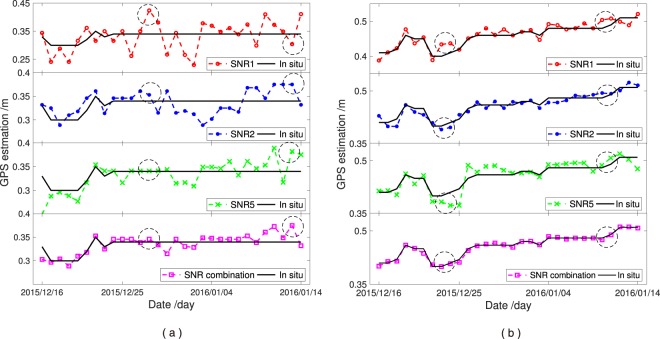
Snow depth estimation of SNR1, SNR2, SNR5 and proposed method based on data collected in the 2015–2016 winter seasons. (**a**) YEL2; (**b**) KIRU.

Snow depth estimations from the SNR1, SNR2, SNR5 and SNR combination data over one month of winter season (2015–2016) at YEL2 and KIRU are used in this study (Fig. 10). The *in situ* snow depth measurements are also shown in the figure. The results indicate that the *in situ* snow depths of the winter season is highly similar to the snow depth estimation based on all four SNR methods in the two sites, thereby showing excellent consistency. However, some differences can still be observed. The performance of the SNR combination method is better than that of the other three methods. An excellent match occurs from the triple-frequency SNR combination method, whereas the estimation error is relatively large for the snow depth estimations of the GPS SNR1, SNR2 and SNR5 observations, according to the results inside the virtual coil shown in Fig. 10. The underestimation percentage can be large because the land surface snow depths around the two GPS sites are small (within half a metre). The underestimation of the snow depth shown in Fig. 10 could be due to the penetration of the L-band signal through the media. Evaluating and comparing the performances of the different methods using *in situ* snow depth measurements at the GNSS station as ground truth in the future are essential. Moreover, snow depth was recorded once a day at a specific time point, whereas snow depth estimation was performed on the basis of the GPS observations over a specific period of several hours. Such a difference may also contribute to the estimation error. Each ascending or descending SNR signal is computed to obtain the reflected height. The accuracy of the reflected heights detected using different carriers or satellites for each day will result in variations. Thus, daily snow depth is regarded as the average values of all depths from each valid SNR trace to diminish the random errors. The random errors in the assumed ground truth are produced when snow depth considerably varies, and they could be the main estimation errors in this case. Therefore, the SNR combination method enhances snow depth estimation because of large valid SNR measurements and few errors in the average snow depths.

## Discussion and Effects

### Comparison of GPS L2 and SNR Combination of GPS Triple-frequency Signals

The SNR combination method can be used to effectively estimate snow depths with an excellent agreement with *in situ* measurements. The daily snow depth at the YEL2 and KIRU sites is the mean of all depths from each valid GPS SNR data. Not all GPS tracks are useful for estimating snow depth, and only those peak amplitudes with several times of the background noise are reserved. The SNR combination method has more SNR observations than GPS single-frequency signals. Thus, the estimations from the SNR combination method are close to the *in situ* snow depths. For snow depths estimated using GPS L1, L2 or L5 SNR, the L2 estimations are the most accurate. Therefore, L2 estimations are described as references, and snow depths from the SNR combination method can be evaluated by comparing them with GPS L2 estimations.

### Effect of Satellite Elevation Angle

**Figure 11:**
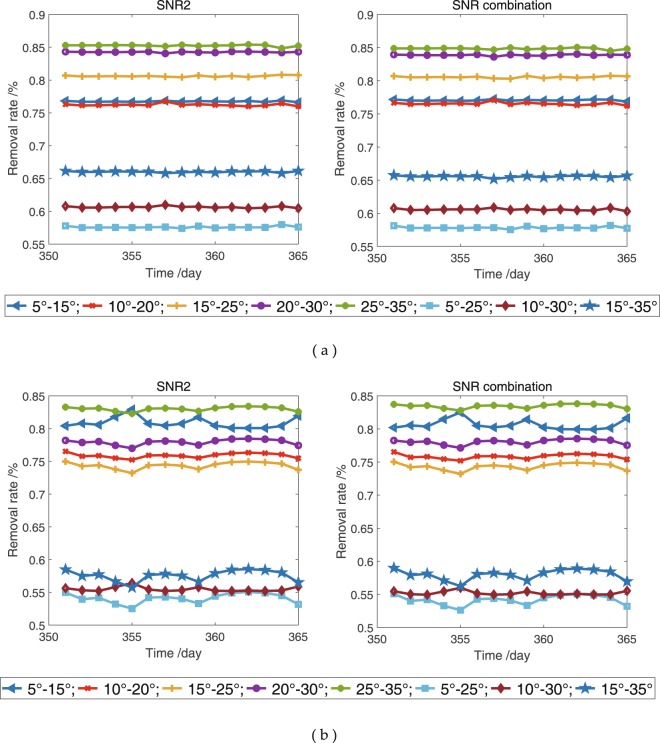
Removal rates of L2 SNR and SNR combination of GPS triple-frequency signals in different satellite elevation coverages. (**a**) YEL2; (**b**) KIRU.

**Figure 12:**
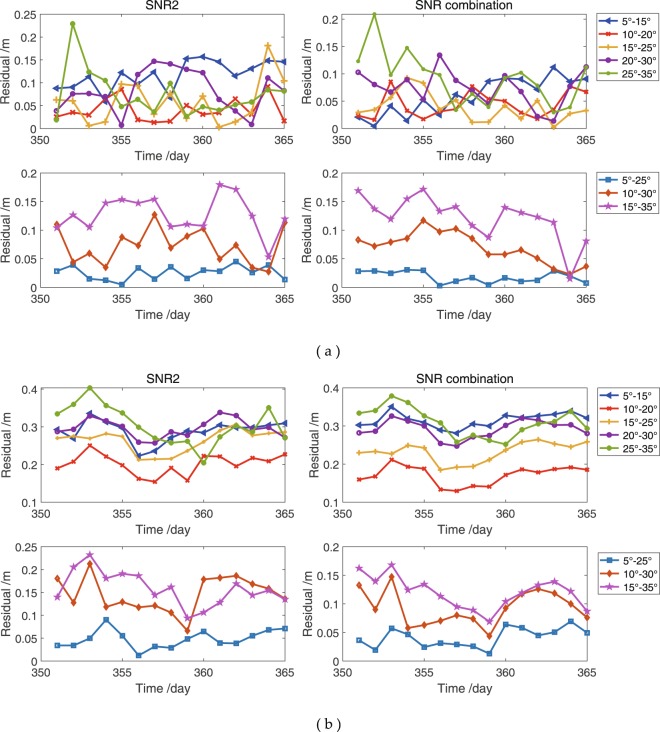
Residuals between estimated snow depths using L2 SNR and SNR combination method and *in situ* snow depths in different satellite elevation coverages. (**a**) YEL2; (**b**) KIRU.

SNR is a key element that affects the accuracy of snow depth estimated on the basis of the GPS-R technique and is essential for the initial SNR data to make a selection at low elevation angles. Elevation angles with different ranges are used to obtain dSNR and estimate snow depths for determining the appropriate range of elevation angles and its effect on snow depth estimation at the YEL2 and KIRU sites. Theoretically, the elevation angle range is determined by oscillations in the SNR series. The elevation angle range, in which range the SNR shows clear sinusoidal or several superimposed oscillations, is considered as the optimal range. However, determining the range in which the SNR shows and does not show clear oscillations is difficult. To date, no specific method exists for the selection of elevation angle range. Elevation angles are usually guessed in low ranges and then refined after post-processing the results. In this study, the elevation angles are divided into eight groups: 5°–15°, 10°–20°, 15°–25°, 20°–30°, 25°–35°, 5°–25°, 10°–30° and 15°–35°. For the initial GPS SNR observations, the relationship ${{\rm{SNR}}}_{{\rm{combination}}} > {{\rm{SNR}}}_{{\rm{L}}2}\ge {{\rm{SNR}}}_{{\rm{L}}1} > {{\rm{SNR}}}_{{\rm{L}}5}$ exists. The SNR combination of GPS triple-frequency signals and L2 SNR for the continuous 15 days in 2015 is randomly selected and treated as an example. According to the above-mentioned elevation angle ranges, the valid SNR data are selected from the initial SNR observations. The removal rate, in which the rate shows the percentage between the valid data and the initial SNR data, is used to evaluate the number of valid SNR observations. The removal rate of SNR data, residuals, ME and RMSE are between the estimated snow depths in different elevation angle coverages and *in situ* snow depths. The results are shown in Figures 11 and 12 and Table 5.

**Table 5:** ME and RMSE between snow depths estimated using L2 SNR and SNR combination and *in situ* measurements in the four ranges of satellite elevation angles.

Station	Observations	Accuracy	10–20°	15–25°	5–25°	10–30°
YEL2	SNR2	ME/cm	4.1	5.8	2.5	7.3
		RMSE/cm	4.8	7.5	2.8	7.9
	SNR combination	ME/cm	4.3	3.9	1.8	7.0
		RMSE/cm	4.9	4.6	2.0	7.4
KIRU	SNR2	ME/cm	15.2	21.3	4.8	9.6
		RMSE/cm	15.3	21.5	5.2	10.0
	SNR combination	ME/cm	12.1	18.2	4.1	9.1
		RMSE/cm	12.3	18.3	4.4	9.7

When the ranges of the elevation angle are 5°–25°, 10°–30° and 15°–35°, the removal rates of SNR are lower than those of the remaining five groups (Fig. 11), and high similar trends are observed for the L2 SNR and SNR combination at the YEL2 and KIRU sites. With the increasing range of elevation angle, the observations are ascending, thereby indicating that several multipath effects will contain substantial height information. Figure 12 shows excellent snow depth estimations from the SNR combination. Snow depths estimated using the SNR data of GPS L2 and the SNR combination with elevation angles from 5° to 25° are the optimal results, with ME values of 1.8 and 4.1 cm and RMSE values of 2.0 and 4.4 cm at the YEL2 and KIRU sites, respectively (Table 5). In addition, the SNR combination-derived snow depths demonstrate slight improvements relative to the L2-derived snow depths at the 5°–25° elevation angles. With the increase in elevation angles, dSNR cannot effectively display the cosine characteristics and obtain an excellent estimation result because of the weak multipath effects. Generally, the effective SNR observations from GPS signals can be maximised in 5°–25° elevation angles and present more evident estimation differences in comparison with the results of the remaining seven groups. Figure 12 shows a strong relationship between snow depth estimations and *in situ* measurements and indicates that snow depths are affected by the increasing range of elevation angles. The estimated snow depths at 5°–25° elevation angles illustrate some deviations because of the significant difference between the GPS station and *in situ* observations. Therefore, a preliminary consideration that the quantity of the SNR data is one of the key elements affecting estimation accuracy is shown.

For the initial SNR data, the appropriate elevation angle range is 5°–25°, and the track that covers the area with the azimuth range is 30°–330° of the GPS receiver in^29^. Therefore, the SNR observations with elevation and azimuth angles of 5°–25° and 30°–330°, respectively, are extracted in the experiment to ensure the integrity of the GPS SNR observations.

**Figure 13:**
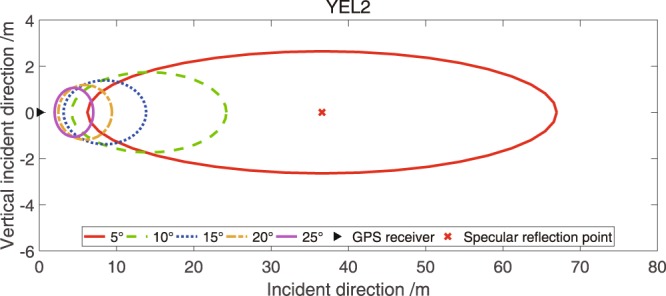
First Fresnel zone for a typical antenna height with different elevation angles. Assuming the GPS L2 (${\rm{\lambda }}=0.244\,{\rm{m}}$) band as an example, the ground antenna height is 1.8 m at YEL2.

The electromagnetic wave covers a certain effective reflection area when reflected on the reflection surface, and the first Fresnel zone is introduced to discuss the effective measurement area. For ground-based GPS observations, the antenna height of the receiver is 1–2 m. The observation surface can be viewed as a plane. The reflection points can be calculated using the elevation and satellite azimuth angles and a simple geometric relationship. The direct wavelength and snow surface are consistent with Fraunhofer theory. Thus, the orthographic projection of the sensing footprint is called the first Fresnel zone at a certain elevation angle. For a surface, the first Fresnel zone can be described as an ellipse, as expressed as follows^4,44^:

7 $$a=\frac{b}{sin\theta };b=\sqrt{\frac{\lambda h}{sin\theta }+{(\frac{\lambda }{2sin\theta })}^{2}},$$

where $\theta$ is the satellite elevation angle, *h* is the antenna height and $\lambda$ is the carrier wavelength. The relationship amongst the first Fresnel zone, satellite elevation angles and antenna height is shown in Fig. 13 on the basis of Equation (7). The centre of the ellipse covered by the specular reflection points (shown as “x”) is $(\frac{\frac{\lambda }{2}+h\,\sin \,\theta }{\sin \,\theta \,\tan \,\theta },0)$, and the projection centre of the antenna is (0, 0). The first Fresnel zone is related to the elevation angle; thus, it changes with the satellite elevation angles. When the effects of the satellite azimuth angles are considered, the reflection area can be regarded as an annulus with a radius of 2a. As described in Fig. 13, the remote sensing footprints become considerably small with the rise in elevation angles; thus, the received reflected signals are small in amplitude. Therefore, the square size of the footprints can be considered the spatial resolution of GNSS-R remote sensing. The specular reflection area is expressed as follows:

8 $${S}={\pi }\mathrm{ab}=\frac{{\rm{\pi }}(4{\lambda }\mathrm{hsin}\theta +{{\lambda }}^{2})}{4{(\sin {\rm{\theta }})}^{3}},$$

### Effect of different snow depths

**Figure 14:**
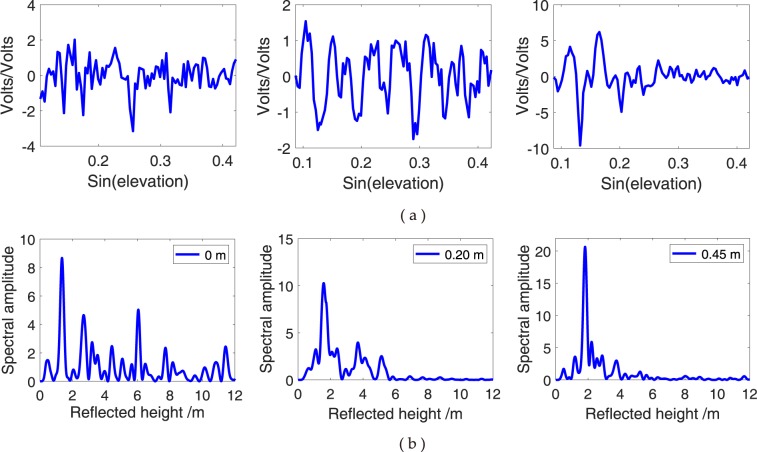
Comparison of SNR combination of GPS triple-frequency signals from YEL2 at different surface snow depths. (**a**) Multipath patterns with different snow depths; (**b**) L–S periodogram of multipath pattern.

**Figure 15:**
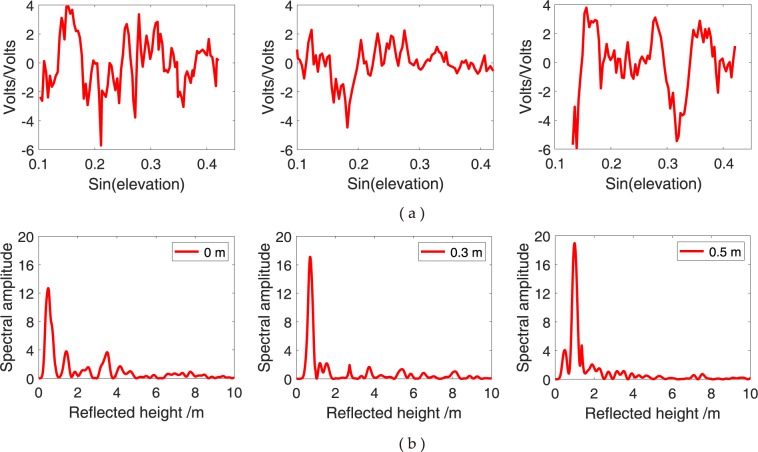
Comparison of SNR combination of GPS triple-frequency signals from KIRU at different surface snow depths. (**a**) Multipath patterns with different snow depths; (**b**) L–S periodogram of multipath pattern.

For multipath reflectometry, the planar surface should be smooth, which is beneficial for coherent reflection. *In situ* snow depths at 0, 0.2 and 0.45 m at YEL2 and 0, 0.3 and 0.5 m at KIRU are selected and compared with the snow depth estimations of the SNR combination method to determine the relationship between different snow depths and SNR combination observations affected by GPS multipath (Figs 14 and 15, respectively). No evident difference is observed for the values of the dSNR of the SNR combination observations with the increase in snow depths. Most of the values are maintained at (−5, 5). In the frequency domain, the frequency oscillation of the dSNR decreases because of the ever-increasing snow depths (Figs 14a and 15a). With the increasing snowfall, the flatness of the snow surface becomes rough, and the strong mirror reflection becomes weak because the diffuse scatter plays a major role when snow depth increases. The peak amplitude gradually decreases with a decrease in GPS antenna height h. This decrease shows a direct relevant relationship with snow depth variations (Figs 14b and 15b). The increase in snow depth makes the peak amplitude move to the left, thereby indicating that the GPS antenna height h becomes small. The results show that the variations of snow depth are closely related to the dynamic changes in the energy spectrum of low-elevation reflection components and produce some regular changes for the GPS SNR observations.

### Comparison of GPS L2 and Triple-frequency SNR Combination

**Figure 16:**
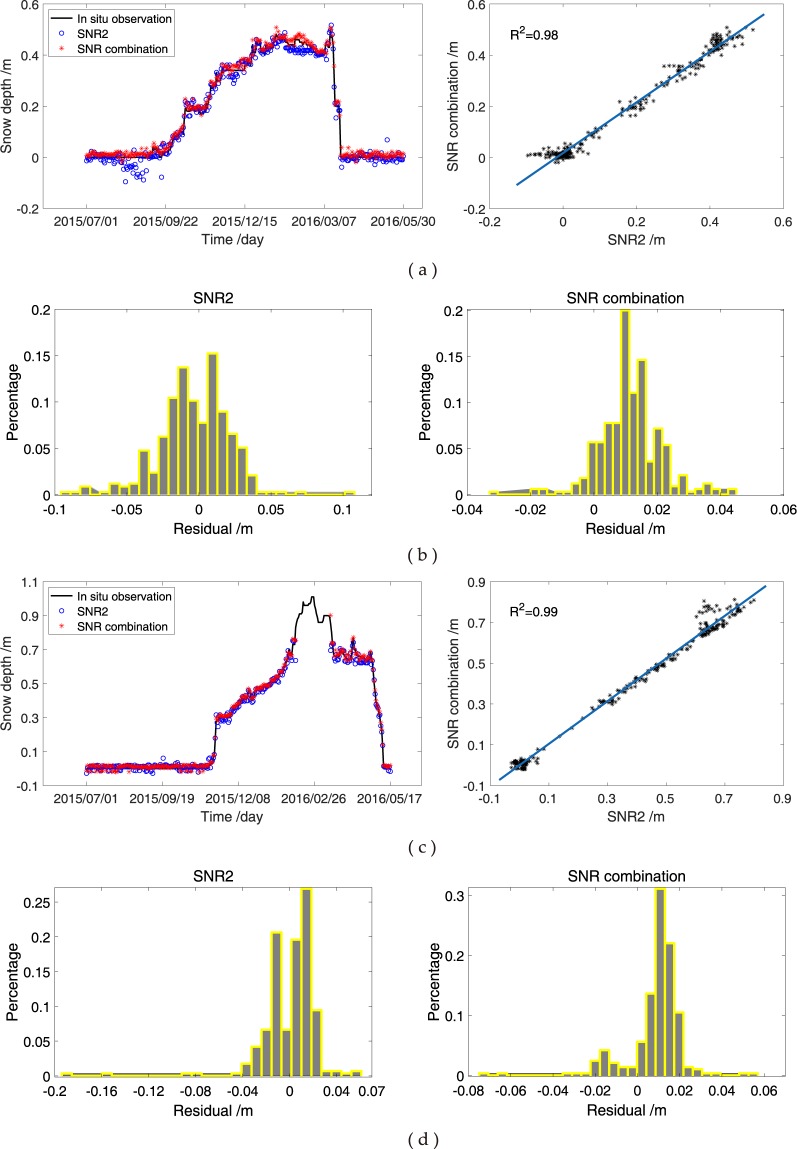
Comparison of GPS L2 SNR and SNR combination of GPS triple-frequency signals. (**a**) Snow depths from GPS L2 and SNR combination and their correlation at YEL2; (**b**) residual percentage from GPS L2 and SNR combination at YEL2; (**c**) snow depths from GPS L2 and SNR combination and their correlation at KIRU; (**d**) residual percentage from GPS L2 and SNR combination at KIRU.

The snow depth estimations of the SNR combination are in excellent agreement with those of L2 SNR at YEL2 and KIRU (Fig. 16a,c, respectively). The correlation coefficients between the L2 SNR and SNR combination are 0.98 and 0.99 at YEL2 and KIRU, respectively. The trend in the winter of 2015 is almost the same for the L2 SNR and SNR combination, and both exhibit a peak height in February 2015. However, the snow depth estimation of the SNR combination is slightly concentrated on the *in situ* measurements. In the sections on Single-frequency SNR Retrievals and Triple-frequency SNR Combination Retrievals, the measurements of 2.6 cm and 1.5 cm for L2-RMSE and SNR combination-RMSE at YEL2, respectively, indicate that the SNR combination method demonstrates a remarkable improvement relative to the GPS L2-estimations. Furthermore, the MEs of the SNR combination and L2 SNR are 1.5 cm and 1.2 cm at YEL2, respectively. The same trends in RMSE and ME are observed at KIRU. Therefore, the SNR combination method can effectively estimate snow depths using geodesic antennas and improve the estimation accuracy of snow depths at GPS stations. The residual percentages of the snow depth estimation of the L2 SNR and SNR combination in the 2015 and 2016 snow seasons are shown and indicate a typical normal distribution trend (Fig. 16b,d, respectively). The residual percentages of the SNR combination in low bias (−2 cm and 2 cm) are far beyond those in high bias.

### Comparison of SNR Combination and Optimal Snow Depth Estimation

**Figure 17:**
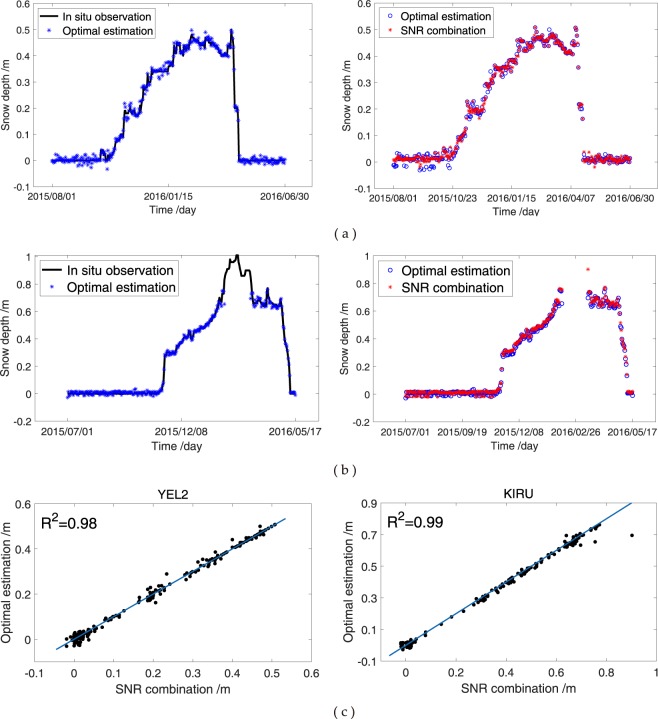
Comparison of SNR combination of GPS triple-frequency signals and optimal estimations from GPS L1, L2 or L5 signals. (**a**) YEL2; (**b**) KIRU; (**c**) correlation coefficients at YEL2 and KIRU.

The results of GPS L2 are slightly worse than those of the SNR combination because insufficient SNR observations result in low accuracy and stability. A new approach using the SNR combination of GPS triple-frequency signals is proposed to improve the accuracy of snow depth estimation from GPS L1, L2 or L5 alone. The optimal estimations (SD_opt_) of the GPS L1-, L2- or L5-derived snow depths are obtained and used as references in accurate estimation to verify the reliability of the proposed approach. The daily optimal snow depth estimations are obtained from the GPS L1-, L2- or L5-derived snow depths at the same day on the basis of the least residuals. The residuals between the snow depth estimations based on the above-mentioned three methods and *in situ* snow depths are obtained. Evidently, SD_opt_ is the daily snow depth estimation with the least residual when making comparisons of the residuals from the above three-mentioned SNR methods. The specific function is

9 $$Re{s}_{Li}=|S{D}_{Li}-S{D}_{obs}|,$$

10 $$\{\begin{array}{c}S{D}_{opt}=S{D}_{L1}\,Re{s}_{L1} < Re{s}_{L2}\,and\,Re{s}_{L1} < Re{s}_{L5}\,\\ S{D}_{opt}=S{D}_{L2}\,Re{s}_{L2} < Re{s}_{L1}\,and\,Re{s}_{L2} < Re{s}_{L5}\,\\ S{D}_{opt}=S{D}_{L5}\,Re{s}_{L5} < Re{s}_{L1}\,and\,Re{s}_{L5} < Re{s}_{L2},\,\end{array}$$

where ${{\rm{SD}}}_{{\rm{L}}1}$, ${{\rm{SD}}}_{{\rm{L}}2}$ and ${{\rm{SD}}}_{{\rm{L}}5}$ are the estimated snow depths from the GPS L1-, L2- and L5-reflected signals, respectively; ${\rm{i}}=[1,\,2,\,5]$; ${{\rm{SD}}}_{{\rm{obs}}}$ is the *in situ* snow depth measurement; and ${{\rm{Res}}}_{{\rm{L}}1}$, ${{\rm{Res}}}_{{\rm{L}}2}$ and ${{\rm{Res}}}_{{\rm{L}}5}$ are the residuals between snow depths from the GPS L1, L2 and L5 signals and the *in situ* measurements, respectively. ${{\rm{SD}}}_{{\rm{opt}}}$ is only regarded as a reference value because this optimal method must acquire *in situ* snow depths in advance. Subpanel labels a and b of Fig. 17 show the comparisons of the SNR combination of GPS triple-frequency (i.e. L1, L2 and L5) signals and optimal estimation of snow depths from the GPS L1, L2 or L5 signals at YEL2 and KIRU for the 2015–2016 snow seasons, respectively. The snow depth estimation of the two methods demonstrates an excellent correlation with the *in situ* observations. In addition, the snow depth estimations from the two methods and the *in situ* observations are considerably smoother with small fluctuations compared with those estimated using the GPS L1, L2 or L5 signals alone. In the snow seasons of 2015–2016, the trends of the SNR combination and optimal estimations are highly similar, with correlation coefficients of 0.98 and 0.99 at YEL2 and KIRU, respectively (Fig. 17c). The correlation and RMSE of the snow depth estimations from the above-mentioned two methods demonstrate no notable differences. Nevertheless, the estimations are better than most snow depths estimated from the GPS L1, L2 or L5 signals alone. These results indicate that the snow depth estimations of the SNR combination show a visible improvement with respect to the existing methods on average. The sensing areas cover a large portion of the antenna with the additional tracks of GPS satellites. Thus, snow reflector heights from different tracks of all satellites also cover additional areas. Further estimations are made daily after converting reflector heights to snow depths in a snow period, which is helpful in estimating abnormal values. When all daily snow depth estimations are averaged, snow depths are considerably close to the *in situ* ones with additional estimations.

### Conclusions

Snow depth variations play an important role in the investigation of global climate. GPS-R is a state-of-the-art detection method that exhibits excellent performance relative to existing techniques for snow depth measurements. Multipath effects appear in GPS measurements due to the interference between direct and reflected signals. The SNR affected by multipath effects can be used to estimate the frequency domains of GPS-reflected signals and reflected height variations. In this study, a new snow depth detection approach is proposed and evaluated using the SNR combination of GPS triple-frequency (i.e. L1, L2 and L5) signals affected by multipath effects at the YEL2 and KIRU sites in Canada and Sweden for one snow season, respectively. The key advantage of the proposed approach with additional SNR observations is that the estimation accuracy is improved by comparing the approach with existing SNR methods. Snow depths can be obtained by using the SNR combination of GPS triple-frequency signals provided by a geodetic receiver, finding the spectral peak frequency of the dSNR time series and using the antenna height of the receiver. The results of the SNR combination method demonstrate an excellent agreement with *in situ* observations, with the correlations being 0.98 and 0.99 at YEL2 and KIRU, respectively. However, the estimation performance of the GPS L1, L2 or L5 signals alone is slightly weaker than that of the SNR combination method. Thus, the accuracy of these SNR methods should be further improved. Furthermore, slight differences between the SNR combination method and the optimal snow depth estimations are found, and the estimations of these methods show a similar trend and correlations of above 0.95 at YEL2 and KIRU. Therefore, snow depths derived from the SNR combination method are as good as the optimal snow depth estimations from the GPS L1, L2 or L5 signals alone. Future work will mainly focus on the snow depth estimation methods using multi-system and multi-frequency GNSS signals due to high spatial resolution with additional satellites. Moreover, the experimental data sets used in this study are limited, and whether the proposed approach demonstrates excellent feasibility at other GNSS stations warrants further investigation.
